# Course of pregnancy and 10-year observation of twins diagnosed with GCK-MODY in the neonatal period: a case report

**DOI:** 10.3389/fendo.2024.1395424

**Published:** 2024-10-01

**Authors:** Barbara Katra, Magdalena Szopa

**Affiliations:** ^1^ Department of Metabolic Diseases, Jagiellonian University Medical College, Kraków, Poland; ^2^ Department of Metabolic Diseases and Diabetology, University Hospital in Krakow, Kraków, Poland

**Keywords:** diabetes, monogenic, MODY, glucokinase, autosomal dominant inheritance

## Abstract

Monogenic diabetes accounts for 5% of all incidence of hyperglycemia and Maturity Onset Diabetes of the Young (MODY) is the most common form. In GCK-MODY, one of the most common forms of MODY, hyperglycemia is caused by a mutation of a gene responsible for coding glucokinase. At the clinical level, this condition presents as persistent, moderate and asymptomatic elevated fasting glucose levels and has a relatively low incidence of micro and macro-vascular complications. In general, the treatment of choice is to follow and maintain a healthy lifestyle. The incidence of GCK-MODY during pregnancy is 2% on average (0-6%). In this report, we introduce a case of a woman diagnosed with GCK-MODY during the pregnancy with twins, a boy and a girl, diagnosed with GCK-MODY after birth. We discuss the course of pregnancy, the need for access to fast and uncomplicated genetic diagnostics *in utero*, and the impact of the MODY diagnosis on the life of the mother and that of her children. In our case, the diagnosis of GCK-MODY was associated with a feeling of relief, after years of uncertainty, and helped to introduce more appropriate eating behaviors and lifestyle changes for both the mother and her children.

## Introduction

Monogenic diabetes accounts for 5% of all incidence of hyperglycemia (1). The most common form of it is Maturity Onset Diabetes of the Young (MODY), which accounts for 1-3% of all cases of diabetes (2–4). Both GCK-MODY (formerly MODY2) together with HNF1A-MODY comprise the most common forms of monogenic diabetes mellitus. The occurrence of GCK-MODY is approximately 1:1000 cases of diabetes (5).

In GCK-MODY, hyperglycemia is caused by a mutation of a gene responsible for coding glucokinase, which is inherited in an autosomal dominant fashion. Glucokinase is an enzyme which transforms glucose into glucose 6-phosphate (G6P) and acts as a sensor for overall glucose levels in blood. As a result of this mutation there is a decreased response of beta cells to glucose level. At the clinical level, this condition presents as persistent, moderate and asymptomatic elevated fasting glucose levels, with an average level of 5.4-8.3 mmol/L (97- 149 mg/dL), HbA1c levels of 6.8-7.5% (40-60mmol/mol), and a 2 hour OGTT (Oral Glucose Tolerance Test) index of <3.0 mmol/l (6, 7).

This disorder begins at fetal life, is detected incidentally during routine examinations, and is passed down from generation to generation. Patients with GCK-MODY have a relatively low incidence of micro and macro-vascular complications (8). Furthermore, it usually does not require pharmacotherapy except in specific cases during pregnancy (9). In general, the treatment of choice is to follow and maintain a healthy lifestyle.

The incidence of GCK-MODY during pregnancy is 2% on average (0-6%) (5, 10–12). In such pregnancies, the occurrence of a typical complication of untreated hyperglycemia, fetal macrosomia, in mothers with GCK-MODY depends on whether or not the child has inherited the mutation. A mutation in the *GCK* gene in the fetus protects it against macrosomia, and subsequently usually does not require insulin therapy for the mother during pregnancy (13, 14). A quick and thorough diagnosis of hyperglycemia in pregnancy makes it possible to apply the appropriate therapeutic management in order to minimize the potential impact of the disorder on both the mother and the fetus.

The diagnosis of GCK-MODY also allows for further management of the patient after pregnancy, as well as for the child and further relatives. Furthermore, it is an indication for an in-depth medical interview in the context of hyperglycemia in the family, as well as a genetic test for the mutation responsible for GCK-MODY, which can explain the cause of potential hyperglycemia discovered during routine periodic examinations.

Here, we introduce a case of a woman diagnosed with GCK-MODY, who was pregnant with twins, a boy and a girl. Biological material from the children was extracted after birth in order to perform genetic testing, the results of which showed the presence of a mutation in the *GCK* gene, which was inherited from the mother, who was also diagnosed with the mutation.

## Case presentation

**Figure 1:**
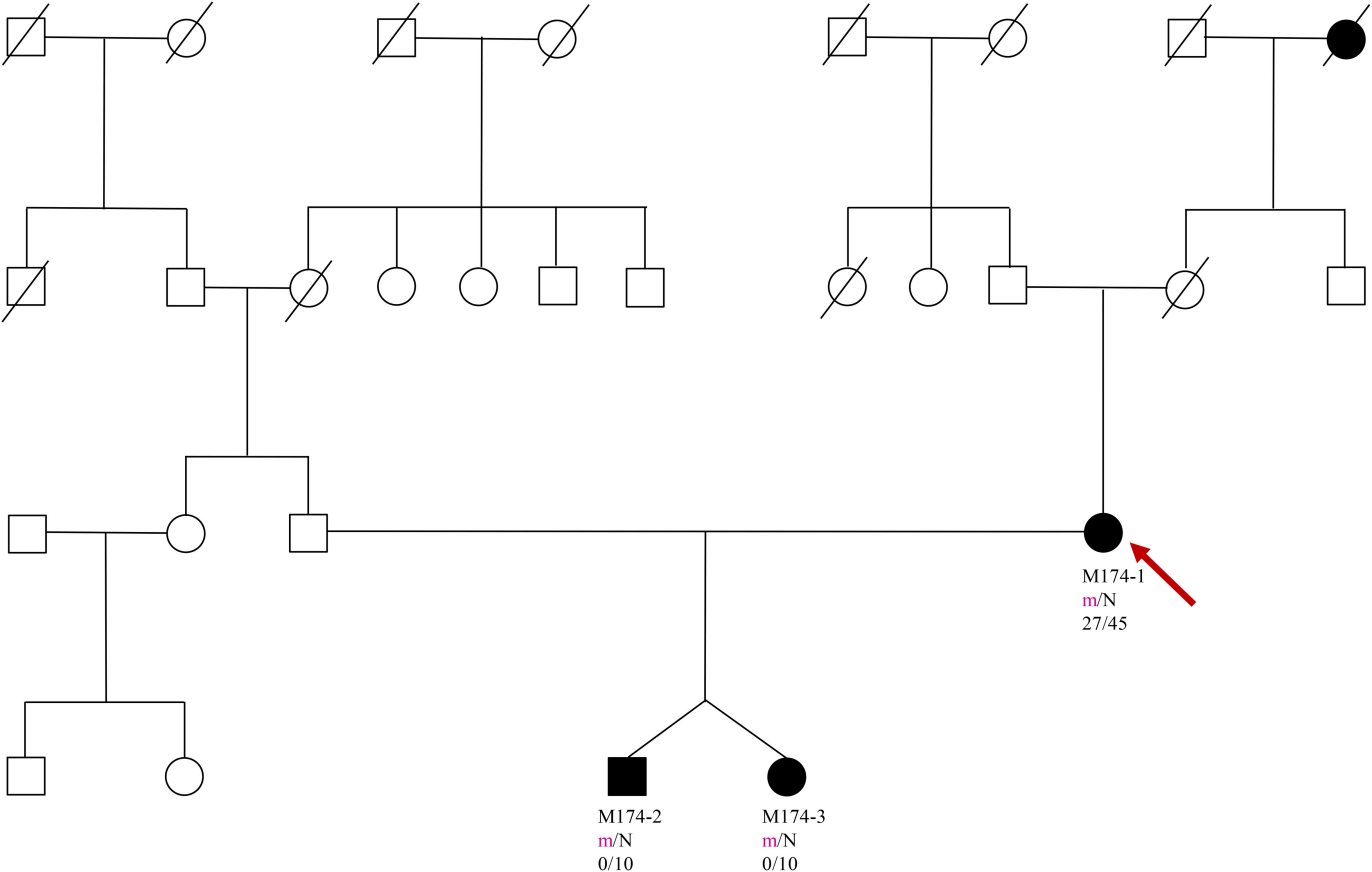
Pedigree, genotypes and clinical characteristics are shown. Segregation of the Phe150del mutation - filled and open symbols represent subjects with hyperglycaemia and normal glucose, respectively. The numbers under the symbols are the identification numbers. Below is the genotype at codon 150: N, normal allele (Phe); m, mutant allele (del). Below the genotype is the age at hyperglycaemia diagnosis for affected members and age at examination. An arrow indicates the index case.

A 35 year old woman in the 6th week of *in vitro* pregnancy presented to the Diabetology Department due to hyperglycemia, with a fasting glycaemia of 125 mg/dL (6.94 mmol/L) observed during the 4th week of pregnancy. The patient relayed a medical history involving an elevated fasting glucose level since she was approximately 27 years old. According to the patient, an OGTT (Oral Glucose Tolerance Test) was performed several times, which revealed abnormal fasting glucose, and a normal glucose level in the 2 hour test. The family history of the patient involves type 2 DM (Diabetes Mellitus) in both the maternal grandmother and great grandmother, with no information on carbohydrate metabolism disorders on the father’s side (**Figure 1**).

The patient’s weight before pregnancy and during the first visit was 57kg, with a height of 160cm and a BMI of 22.3 kg/m2. The patient did not report any other disease. A series of biochemical tests were performed as standard procedure for a first visit to the Diabetology Department, which showed an Hb1Ac of 5.1% (32 mmol/mol) a C-Peptide level of 0.49 ng/mL, and a negative result for anti-GAD antibodies. The patient was instructed to follow a diabetic diet of 1800kcal for pregnant women and to self-monitor using a glucometer at home. The recommended fasting blood glucose was set at 90 mg/dL (5.0 mmol/L), and a 1 hour post-prandial level of 120 mg/dL (6.7 mmol/L). The adjustment criteria is in accordance with the recommendation of the Polish Diabetes Association at the time. Due to a TSH level of 3.91 mIU/L, a gestational subclinical hypothyroidism was diagnosed, and treatment was commenced using levothyroxine. Due to the observed stable fasting hyperglycemia over many years, and a positive family history of diabetes, a blood sample was collected for genetic testing under suspicion of GCK-MODY.

The patient remained in the care of the diabetes and gynecology outpatient clinic, with planned visits every few weeks, and she complied with the rules of the diabetic diet. During the 7th week of pregnancy, it was decided to start intensive insulin therapy due to abnormal fasting and postprandial glucose levels despite initial treatment. The treatment plan consisted of mealtime injections of human insulin and one injection of NPH insulin. At the 9th week, a twin pregnancy was diagnosed on the basis of an ultrasound examination. During subsequent visits to the Diabetology Clinic, an additional injection of NPH insulin was introduced during the morning due to continued elevated blood glucose levels in the afternoon and evening. At the 31st week of pregnancy, the NPH insulin was substituted in favor of detemir insulin. The total daily insulin requirement at 36 weeks was 108 units, with no hypoglycemia. Throughout the pregnancy, the patient’s weight gain was 29kg – data presented in **Table 1**. Throughout the pregnancy, the gynecologist monitored fetal weight (EFW) and abdominal circumference (AC) using ultrasound. At 28 weeks, the EFW of first fetus was 1277g with an AC of 28 + 6 and the second 1187g with an AC of 28 + 2.

**Table 1:** Insulin doses of the patient and fetal parameters (Estimated Fetel Weight – EFW, Abdominal Circumference – AC) in the course of pregnancy.

Week of pregnancy	Body mass [kg]	Basal insulin [units]	Prandial Insulin [units]	Daily dose of insulin [units]	Fetus I EFW[g] AC (cm)	Fetus II EFW[g] AC (cm)
**6**	57	6	13	29		
**9**		6	13	19		
**12**	59.5	6	15	21		
**18**	65	7	15	22		
**23**	70	7	17	24		
**26**		12	28	40		
**28**	75	25	46	71	1277 28+6 (AC)	1187 28+2 (AC)
**31**	77	30	70	100	2000	2100
**36**	86	26	82	108		

Shortly before birth, genetic testing confirmed a diagnosis of GCK-MODY in the mother. A heterozygous pathogenic variant was detected at c.[449_451del];[=], p.[(Phe150del),(=)] in the *GCK* gene. A Caesarean section was performed at the 36th week of pregnancy. The birth weight for the girl was 2200g and the boy was 2300g. The children were born healthy without hypoglycemia after birth. With the mother’s consent, blood samples were taken from the children for genetic testing immediately after birth (at that time, it was not possible to genetically test children before birth). The testing confirmed the inheritance of the same pathogenic variant of the *GCK* gene in both children. Other family members showed no interest in genetic testing.

10 years after delivery, the patient weighs 57kg and has since been diagnosed with hypertension, which is well controlled using amlodipine. Blood glucose levels during self-monitoring using a glucometer show morning fasting blood glucose levels of 125-140 mg/dL (6.97-7.77 mmol/L), and post-prandial levels of 130-135 mg/dL (7.21-7.49 mmol/L). In order to obtain accurate measurements, glucose monitoring was performed using the Free Style Libre 2 system, with a sensor activity time of 91% and TIR (Time in Range) of 98% and TAR (Time Above Range) of 2%. The average glucose concentration was 134 mg/dL (7.44 mmol/L).

As of the time of writing, the children are 10 years old. The son weights 25kg (approx. 10th percentile) with a height of 134,5cm (approx. 50th percentile) and the daughter weighs 23kg (approx. 10th percentile) with a height of 134,5cm (approx. 50th percentile). Intellectual assessment through development and learning is very good, and the children are currently not under any glycemic control.

A survey was conducted with the patient, regarding the impact of the MODY diagnosis on her life on that of her children – outlined in **Table 2**. The patient stated that the diagnosis of GCK-MODY helped explain the cause of her fasting hyperglycemia, and changed her life to some extent. She added that she felt relieved that she would not have to use insulin therapy for the type of diabetes she was diagnosed with. The patient was prompted to quickly perform genetic tests for her children in order to diagnose the cause of potential hyperglycemia and to implement the appropriate management, which involves a diet with limited sugar and an active lifestyle. Additionally, the patient was also asked to complete a Problem Areas in Diabetes questionnaire (PAID-5). This questionnaire is a validated self-reporting tool measuring diabetes distress, and is widely used in both clinical and research settings (15). It contains 20 items, asking about problem areas such as being scared of living with diabetes, feelings of deprivation regarding food and meals, and worrying about low blood sugar reactions (16). PAID uses a five-point Likert scale to assess the response to each item. The total score ranges from 0-100, achieved by summing up 0-4 responses to 20 PAID items, and multiplying the sum by 1.25, with higher scores indicating higher levels of distress (16). Elevated diabetes distress is arbitrarily defined with a total score of ≥ 40, which roughly equates to 1SD above the mean (17). The patient scored 30 points on her questionnaire. She highlighted 3 categories as fairly serious problems (3 points on the Likert scale): Feeling discouraged about her treatment plan, the feeling of losing the ability to eat certain foods, and worrying about the future and possibility of serious complications. Her moderate concerns (2 points on the Likert scale) involved feeling constantly concerned about food and eating, feeling guilty or afraid when not dealing with diabetes, and not coming to terms with diabetes. Nine items were considered minor problems, and five items were no problem. The questionnaire as completed by the patient can be seen in **Table 3**.

**Table 2:** Questionnaire with responses by the patient.

Question	Answer
Has been diagnosed with MODY diabetes changed your life, and how?	To a small extent, beyond the general awareness of what I suffer from, and what lifestyle I should lead. Making the diagnosis finally answered the question of why I have high fasting sugar.
Has being diagnosed with MODY diabetes changed your perception of diabetes, and how?	In a real sense, I was relieved that I have a specific type of diabetes that does not require insulin treatment, but require discipline in everyday life.
What prompted you to perform the tests for you children so quickly?	The ability to conduct research as part of scientific research and to build awareness. From the beginning, I wanted to raise the children properly and make the family and educational institutions aware of their condition.
How did the diagnosis of MODY diabetes in your children affect your behavior towards them (ie frequent checkups, visits to the doctor)?	First of all, it allowed me to create a disciplined environment in terms of diet. A fairly strict diet was introduced from the beginning, and sweets were only introduced around the age of 3. Visits to the doctor were not more frequent than usual, because the children rarely get sick in general.
How has the diagnosis of MODY diabetes in the children affected their lives?	Primarily in the context of their lifestyle. We are active in sports, and watch our diet, and the children are aware that they have diabetes

**Table 3:** Problem Areas In Diabetes (PAID) Scale filled out by the patient.

		0	1	2	3	4
1	Not having clear and concrete goals for your diabetes care?	X				
2	Feeling discouraged with your diabetes treatment plan?				X	
3	Feeling scared when you think about living with diabetes?		X			
4	Uncomfortable social situations related to your diabetes care (e.g. people telling you what to eat)?	X				
5	Feelings of deprivation regarding food and meals?				X	
6	Feeling depressed when you think about living with diabetes?		X			
7	Not knowing if your mood or feelings are related to your diabetes?		X			
8	Feeling overwhelmed by your diabetes?		X			
9	Worrying about low blood glucose reactions?		X			
10	Feeling angry when you think about living with diabetes?		X			
11	Feeling constantly concerned about food and eating?			X		
12	Worrying about the future and the possibility of serious complications?				X	
13	Feelings of guilt or anxiety when you get off track with your diabetes management?			X		
14	Not accepting your diabetes?			X		
15	Feeling unsatisfied with your diabetes physician?	X				
16	Feeling that diabetes is taking up too much of your mental and physical energy every day?		X			
17	Feeling alone with your diabetes?	X				
18	Feeling that your friends and family are not supportive of your diabetes management efforts?	X				
19	Coping with complications of diabetes?		X			
20	Feeling burned out by the constant effort needed to manage diabetes?		X			

Instructions: Which of the following diabetes issues are currently a problem for you? Tick the box that gives the best answer for you. Please provide an answer for each question. “X” is the Patient selected answer.

## Discussion

Making the right diagnosis using molecular testing at the appropriate time remains a challenge, and it is very important in order to choose the appropriate therapy for the patient, in order to optimize their comfort and the comfort of their family members (18). However, the situation in which fraternal twins were diagnosed with GCK-MODY shortly after birth has not been described in literature so far. This provided a unique opportunity to prospectively observe their development, and assess the impact of the GCK-MODY diagnosis on their lives and their families’.

For the described patient, the diagnosis of GCK-MODY was associated with a feeling of relief, resulting from a concrete diagnosis after years of uncertainty, as well as from implementing the appropriate management for her condition. According to the patient, it was effective in motivating her family to engage in more appropriate eating behaviors. The children were made aware of the diagnosis, and accordingly had their lifestyles adjusted.

Given the nature of screening for hyperglycemia in pregnancy, there is an increased possibility of discovering monogenic diabetes in women, and by extension their genetic family. A proper diagnosis of this type of diabetes in pregnancy is also very important for the application of appropriate management during pregnancy. According to the results of The Atlantic Diabetes in Pregnancy Cohort, the incidence of the pathogenic variant in *GCK* in women with GDM is 0.1% (5). The authors of this study formulated a set of criteria for performing genetic testing for GCK-MODY for Caucasians. Pregnant women with a normal pre-pregnancy BMI (<25kg/m2) and a fasting of blood glucose of ≥ 99 mg/dL (5.5 mmol/L) in the blood sugar curve should be tested for the presence of a pathogenic variant in the *GCK* gene. Such diagnostic criteria are economical, and allow for the detection one case of GCK-MODY in pregnancy via genetic testing of 2.7 patients with GDM. Our patient met the criteria suggesting GCK-MODY well, as she had elevated fasting glucose, slim body weight and did not develop hypoglycemia during pregnancy despite relatively high doses of insulin.

Confirmation of the presence of a pathogenic variant in the *GCK* gene in the fetus does not necessarily require the introduction of insulin therapy during pregnancy (19). It is therefore very important to develop the ability to conduct genetic tests without risk to the fetus, as was the case for our patient, wherein genetic testing was only possible immediately after birth. Quick and ready access to diagnostic methods would allow for a diagnosis in a pregnant woman and then in the offspring before the end of pregnancy, which in turn would allow to avoid the use of insulin in the mother during pregnancy. In the case of our patient, the daily requirement for insulin in the 36th week of pregnancy was 108 units. An alternative to this procedure would have been to introduce insulin therapy in the mother in the case of too-rapid growth of the fetuses, based on ultrasound examination in accordance with the recommendations in year of 2015 (10). The next step in diagnosing monogenic diabetes in a pregnant or planning-to-be-pregnant woman should be a proper consultation in the field of diabetes genetics.

According to recommendations by the ADA, diabetes related distress should also be defined. The recommended tool for this purpose is the PAID-5 questionnaire, which was used in this study. Our patient scored 30 points, which puts her at risk of moderate distress related to diabetes (17-39 points) (20). There is a significant subgroup of people with diabetes that express moderate levels of diabetes distress, with a risk of increasing over time, which may benefit from early detection and preventive measures (20). It is also worth noting that women tend to report higher diabetes distress compared to men (17), and younger people are also more likely to report elevated diabetes distress (21). For example, in the 2nd edition DAWN study, the average score obtained by patients from Poland with diabetes was 41.6. A score of ≥40, indicating acute distress related to diabetes, was obtained by 56.7% of respondents in Poland, taking 14th place in the world. The results obtained in Poland were among the four least favorable in the world. This result did not differ significantly from the first edition of the DAWN study, conducted 10 years earlier. The Polish results indicated a relatively low well-being of diabetic patients, despite having a relatively good sense and knowledge base of the disease. The analysis of the PAID-5 questionnaire for our patient definitively facilitated the individualization of therapy and education for both her and her children.

## Conclusions

The described situation demonstrates the need for vigilance by physicians treating hyperglycemia in pregnancy. Dissemination of the diagnostic algorithm according to The Atlantic Diabetes in Pregnancy Cohort will allow physicians to discover more people with the glucokinase gene mutation. Of course, this must be supported by access to fast and uncomplicated genetic diagnostic. The psychological aspect of this diagnosis is also crucial: genetic diagnosis can provide a sense of relief and help guide treatment, but it can also bring additional concerns, which should be properly detected and supported with adequate education and support.

## Data Availability

The original contributions presented in the study are included in the article, further inquiries can be directed to the corresponding author/s.
